# Supplementary material for: West African–South American pandemic Vibrio cholerae encodes multiple distinct phage defence systems
Source: Nat Microbiol. 2025 May 22;10(6):1352–65. doi: 10.1038/s41564-025-02004-9 (PMC12137116; doi:10.1038/s41564-025-02004-9)
Supplement: Supplementary file 1 — Legends for Supplementary Tables 1–16, Supplementary Figs. 1–3 and Supplementary References. [file 41564_2025_2004_MOESM1_ESM.pdf]

nature microbiology

Supplementary information

https://doi.org/10.1038/s41564-025-02004-9

West African–South American pandemic *Vibrio cholerae* encodes multiple distinct phage defence systems

In the format provided by the authors and unedited

Contents:

Legends for Supplementary Tables 1-16
Legends for Supplementary Videos 1-2
Supplementary Figures 1-3
Supplementary References

Supplementary Tables

Table 1. Summary of defence systems identified by DefenseFinder and PADLOC

Table 2. Summary of WASA-1 hits detected by BLAST in the genus *Vibrio*

Table 3. Summary of defence systems identified in WASA-1 hits by DefenseFinder / PADLOC

Table 4. Summary of matching hits for the *Vibrio cholerae* WonAB (A1552VC_01233-34) model, detected by MacSyFinder v.2.1.1

Table 5. Summary of matching hits for the *Anaerovibrio lipolyticus* OLD-ABC ATPase + Novel REase (SAMN02745671_01839-38) model, detected by MacSyFinder v.2.1.1

Table 6. Summary of matching hits for the *Vibrio cholerae* GrwAB (A1552VC_00274-75) model, detected by MacSyFinder v.2.1.1

Table 7. Summary of matching hits for the *Vibrio cholerae* *Vc*SduA (A1552VC_00276) model, detected by MacSyFinder v.2.1.1

Table 8. Summary of matching hits for the *Vibrio cholerae* GrwAB-*Vc*SduA (A1552VC_00274-75-76) model, detected by MacSyFinder v.2.1.1

Table 9. Summary of DpdA-encoding vibriophages detected by BLAST

Table 10. *V. cholerae* strains used in this work

Table 11. *E. coli* strains used in this work

Table 12. Plasmids used in this work

Table 13. DNA oligonucleotides used for the construction of plasmids and strains

Table 14. Vibriophages used in this work

Table 15. BASEL collection bacteriophages used in this work

Table 16. Defence systems identified in this work

Supplementary Videos

Supplementary Video 1. Time-lapse microscopy of ICP1 infection.

Time-lapse microscopy comparing exponentially growing cells of *V. cholerae* A1552 WT and ΔWASA-1 strains after infection with ICP1-2006 at MOI 5. Cells were grown at 37°C on LB agarose and imaged automatically at 1 minute intervals for 60 minutes. Bar = 20 µm. Playback = 5 fps.

Supplementary Video 2. Time-lapse microscopy of Bas24 infection.

Time-lapse microscopy comparing exponentially growing cells of *E. coli* MG1655Δ*araCBAD* in the absence (No system) and presence of GrwAB (+ GrwAB) production, after infection with Bas24 at MOI 10. Cells were grown at 37°C on LB agarose + 5mM $CaCl_2$, 20mM $MgSO_4$, 0.2% arabinose and imaged automatically at 1 minute intervals for 180 minutes. Bar = 20 µm. Playback = 5 fps.

Supplementary Figures

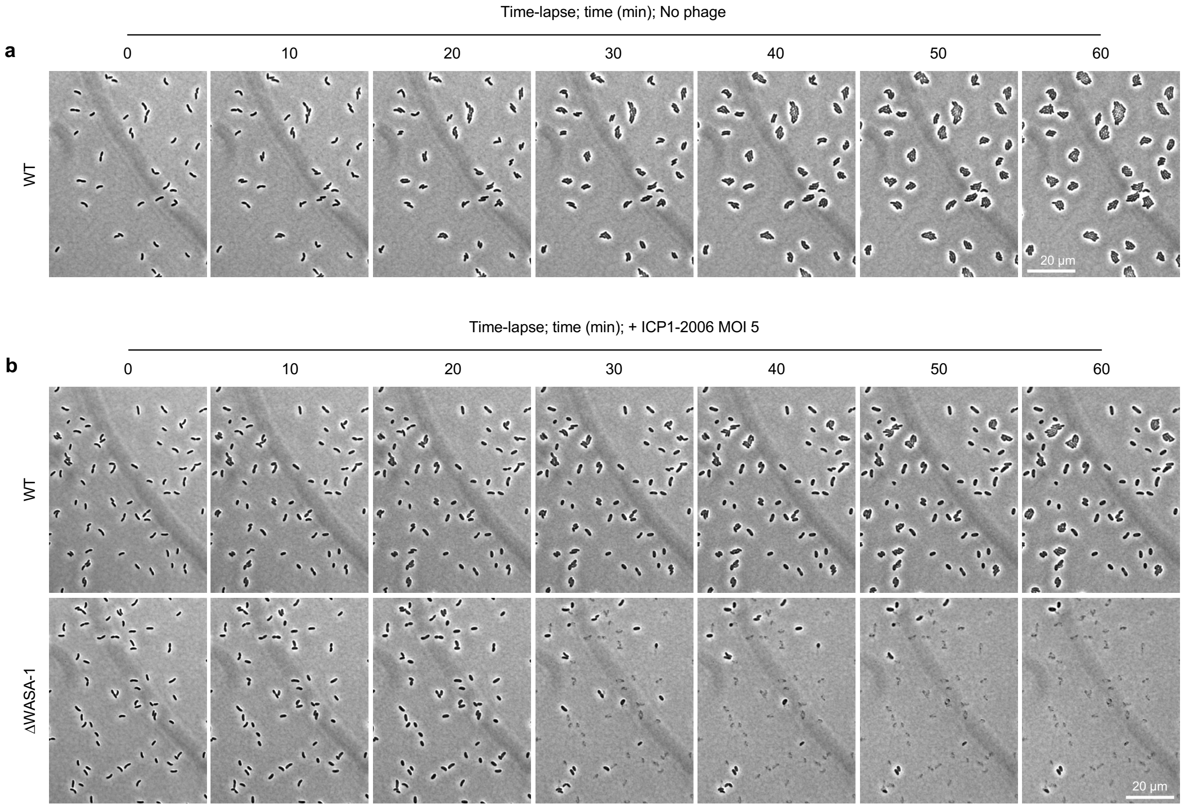

Supplementary Fig. 1. Time-lapse microscopy of ICP1 infection. (**a**) Control experiment showing time-lapse microscopy of exponentially growing cells of *V. cholerae* A1552 (WT) in the absence of phage infection. (**b**) Time-lapse microscopy comparing exponentially growing cells of *V. cholerae* WT and ΔWASA-1 strains after infection with ICP1-2006 at MOI 5. The panels depict the full-frame versions of the examples presented in Fig. 2b. All images are representative of the results of three independent experiments. Scale bars = 20 µm.

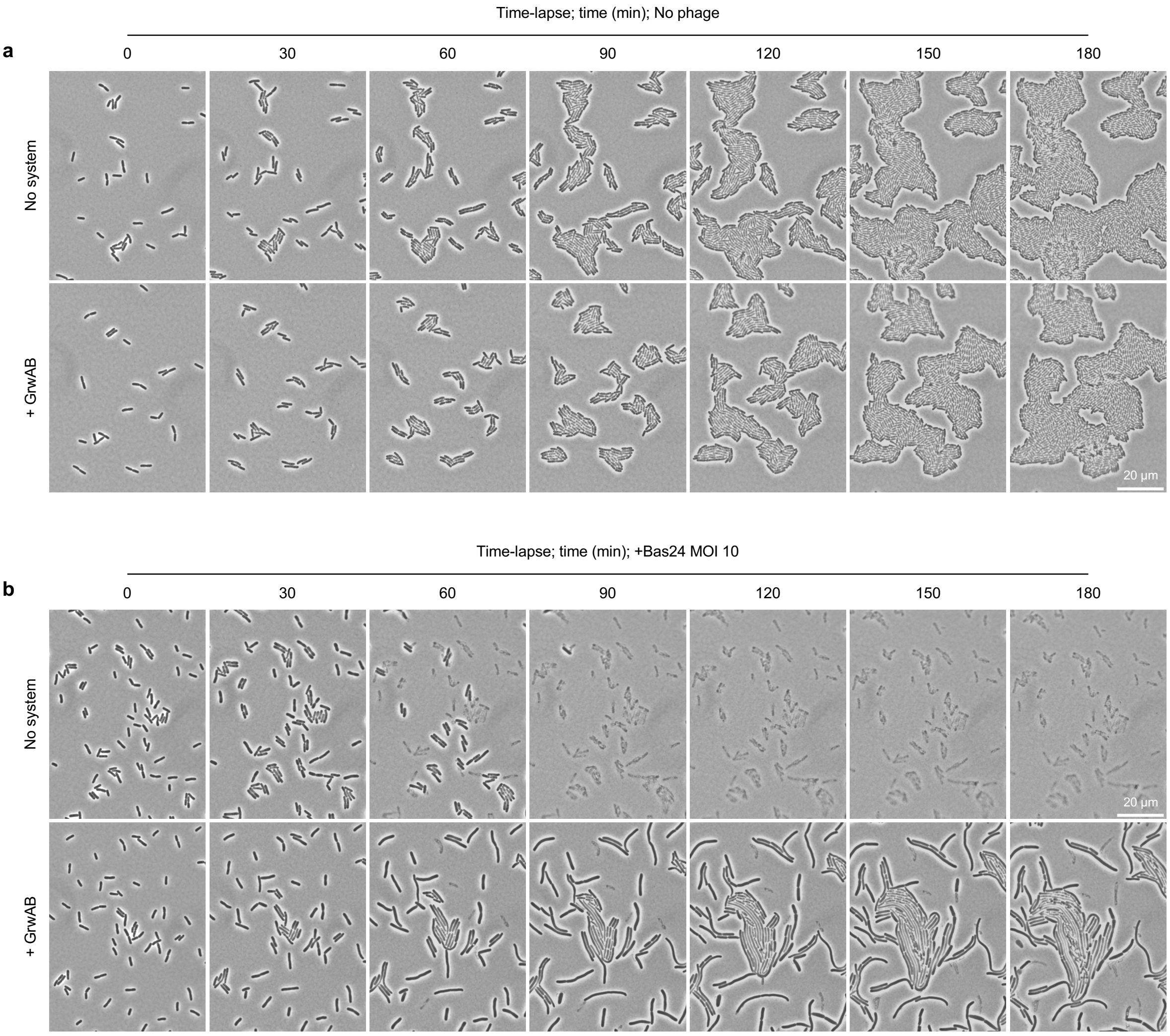

Supplementary Fig. 2. Time-lapse microscopy of Bas24 infection. (**a**-**b**) Time-lapse microscopy of exponentially growing cells of *E. coli* MG1655Δ*araCBAD* in the absence (No system) and presence (+ GrwAB) of GrwAB production, in the absence (**a**) and presence (**b**) of infection with Bas24 at MOI 10. All images are representative of the results of three independent experiments. Scale bars = 20 µm.

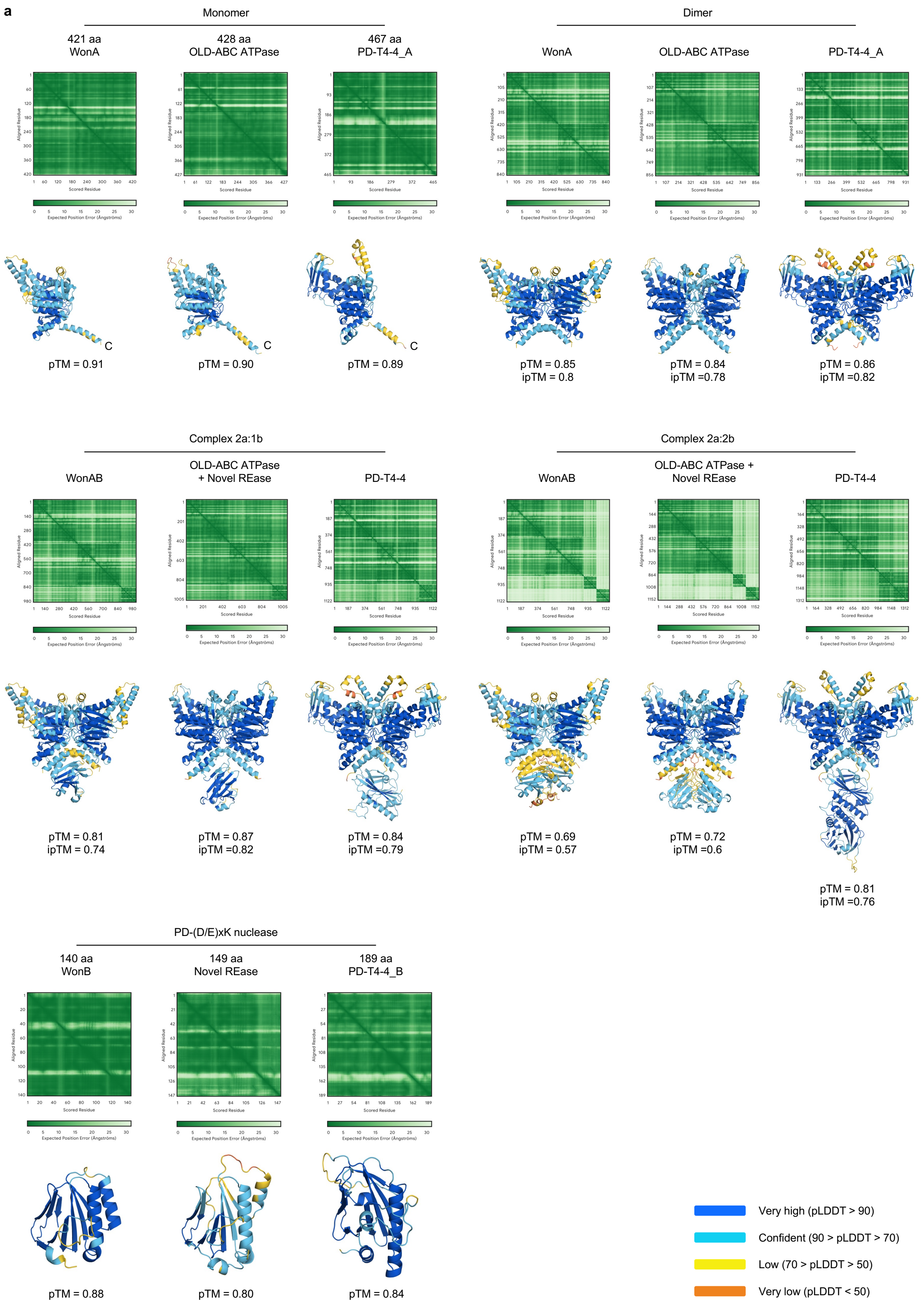
a
Monomer
421 aa
WonA
428 aa
OLD-ABC ATPase
467 aa
PD-T4-4_A
Dimer
WonA
OLD-ABC ATPase
PD-T4-4_A
C
pTM = 0.91
pTM = 0.90
pTM = 0.89
pTM = 0.85
ipTM = 0.8
pTM = 0.84
ipTM =0.78
pTM = 0.86
ipTM =0.82
Complex 2a:1b
WonAB
OLD-ABC ATPase
+ Novel REase
PD-T4-4
Complex 2a:2b
WonAB
OLD-ABC ATPase +
Novel REase
PD-T4-4
pTM = 0.81
ipTM = 0.74
pTM = 0.87
ipTM =0.82
pTM = 0.84
ipTM =0.79
pTM = 0.69
ipTM = 0.57
pTM = 0.72
ipTM =0.6
pTM = 0.81
ipTM =0.76
PD-(D/E)xK nuclease
140 aa
WonB
149 aa
Novel REase
189 aa
PD-T4-4_B
pTM = 0.88
pTM = 0.80
pTM = 0.84
Aligned Residue
Scored Residue
Expected Position Error (Ångströms)
Very high (pLDDT > 90)
Confident (90 > pLDDT > 70)
Low (70 > pLDDT > 50)
Very low (pLDDT < 50)

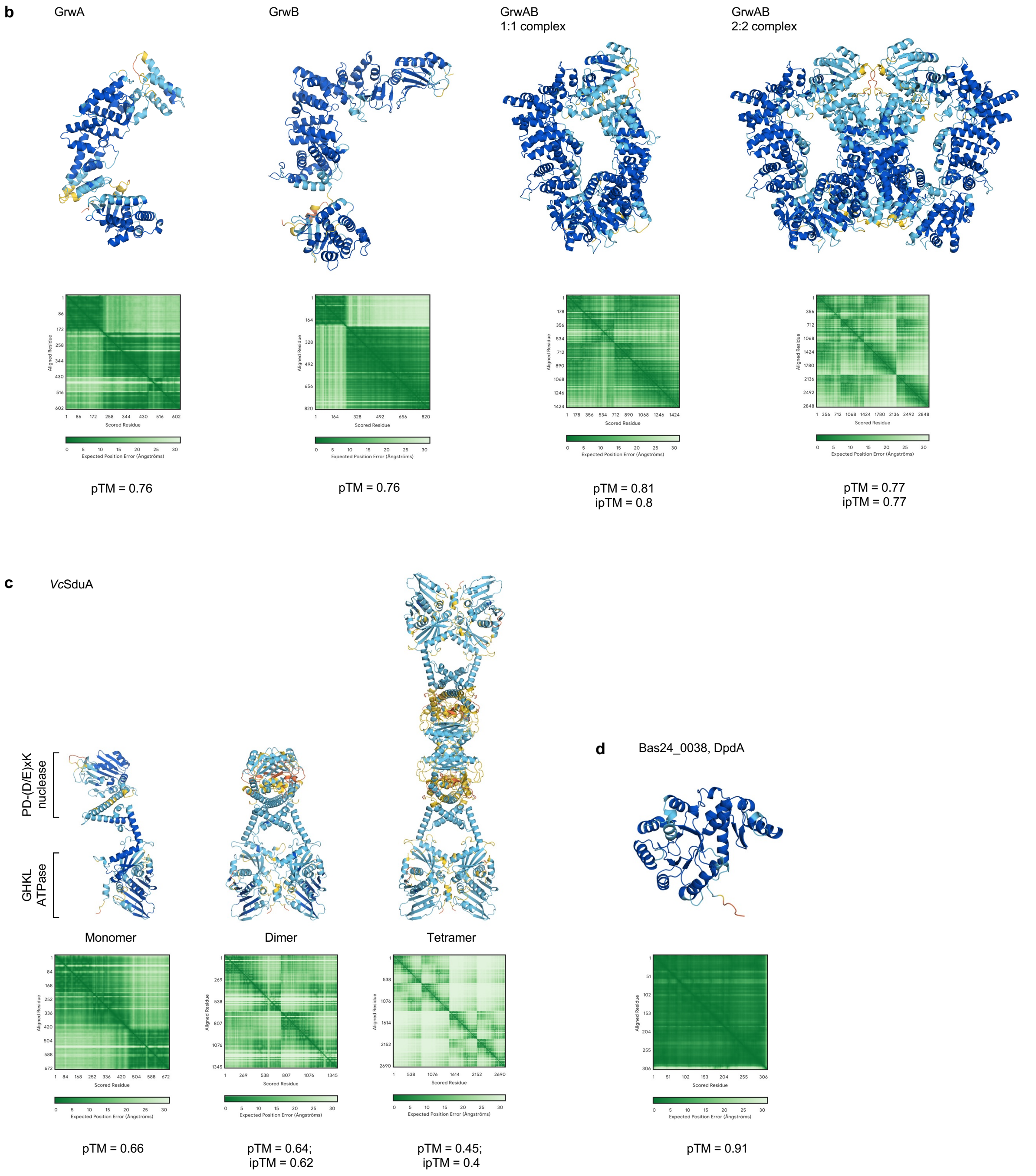

Supplementary Fig. 3. Confidence metrics for AlphaFold3 models.

AlphaFold3 models of (**a**) WonAB and related systems (**b**) GrwAB, (**c**) *Vc*SduA and (**d**) DpdA are shown below predicted aligned error (PAE) plots and are coloured according to per residue predicted local distance difference test (pLDDT) score. The predicted template modelling (pTM) score, and where appropriate the interface predicted template modelling (ipTM) score, are shown below each model.

Supplementary References

1 Chattopadhyay, D. J. *et al.* New phage typing scheme for *Vibrio cholerae* O1 biotype El Tor strains. *J Clin Microbiol* **31**, 1579-85, doi:10.1128/jcm.31.6.1579-1585.1993 (1993).

2 Heidelberg, J. F. *et al.* DNA sequence of both chromosomes of the cholera pathogen *Vibrio cholerae*. *Nature* **406**, 477-83, doi:10.1038/35020000 (2000).

3 Pearson, G. D., Woods, A., Chiang, S. L. & Mekalanos, J. J. CTX genetic element encodes a site-specific recombination system and an intestinal colonization factor. *Proc Natl Acad Sci U S A* **90**, 3750-4, doi:10.1073/pnas.90.8.3750 (1993).

4 Miller, V. L., DiRita, V. J. & Mekalanos, J. J. Identification of *toxS*, a regulatory gene whose product enhances *toxR*-mediated activation of the cholera toxin promoter. *J Bacteriol* **171**, 1288-93, doi:10.1128/jb.171.3.1288-1293.1989 (1989).

5 Yildiz, F. H. & Schoolnik, G. K. Role of *rpoS* in stress survival and virulence of *Vibrio cholerae*. *J Bacteriol* **180**, 773-84, doi:10.1128/JB.180.4.773-784.1998 (1998).

6 Stutzmann, S. & Blokesch, M. Comparison of chitin-induced natural transformation in pandemic *Vibrio cholerae* O1 El Tor strains. *Environ Microbiol* **22**, 4149-66, doi:10.1111/1462-2920.15214 (2020).

7 Wachsmuth, I. K. *et al.* The molecular epidemiology of cholera in Latin America. *J Infect Dis* **167**, 621-6, doi:10.1093/infdis/167.3.621 (1993).

8 Blokesch, M. TransFLP-a method to genetically modify *Vibrio cholerae* based on natural transformation and FLP-recombination. *J Vis Exp*, doi:10.3791/3761 (2012).

9 Blokesch, M. & Schoolnik, G. K. Serogroup conversion of *Vibrio cholerae* in aquatic reservoirs. *PLoS Pathog* **3**, e81, doi:10.1371/journal.ppat.0030081 (2007).

10 Vizzarro, G., Lemopoulos, A., Adams, D. W. & Blokesch, M. *Vibrio cholerae* pathogenicity island 2 encodes two distinct types of restriction systems. *J Bacteriol*, e0014524, doi:10.1128/jb.00145-24 (2024).

11 Meyer, N., Stephan, R., Cernela, N., Horlbog, J. A. & Biggel, M. Genomic characteristics of clinical non-toxigenic *Vibrio cholerae* isolates in Switzerland: a cross-sectional study. *Swiss Med Wkly* **154**, 3437, doi:10.57187/s.3437 (2024).

12 Matthey, N., Drebes Dörr, N. C. & Blokesch, M. Long-Read-Based Genome Sequences of Pandemic and Environmental *Vibrio cholerae* Strains. *Microbiol Resour Announc* **7**, doi:10.1128/MRA.01574-18 (2018).

13 Simon, R., Priefer, U. & Pühler, A. A Broad Host Range Mobilization System for In Vivo Genetic Engineering: Transposon Mutagenesis in Gram Negative Bacteria. *Bio/Technology* **1**, 784-91, doi:10.1038/nbt1183-784 (1983).

14 Ferrières, L. *et al.* Silent mischief: bacteriophage Mu insertions contaminate products of *Escherichia coli* random mutagenesis performed using suicidal transposon delivery plasmids mobilized by broad-host-range RP4 conjugative machinery. *J Bacteriol* **192**, 6418-27, doi:10.1128/JB.00621-10 (2010).

15 Aoki, S. K. *et al.* A universal biomolecular integral feedback controller for robust perfect adaptation. *Nature* **570**, 533-37, doi:10.1038/s41586-019-1321-1 (2019).

16 De Souza Silva, O. & Blokesch, M. Genetic manipulation of *Vibrio cholerae* by combining natural transformation with FLP recombination. *Plasmid* **64**, 186-95, doi:10.1016/j.plasmid.2010.08.001 (2010).

17 Van der Henst, C. *et al.* Molecular insights into *Vibrio cholerae*'s intra-amoebal host-pathogen interactions. *Nat Commun* **9**, 3460, doi:10.1038/s41467-018-05976-x (2018).

18 Metzger, L. C. *et al.* Independent Regulation of Type VI Secretion in *Vibrio cholerae* by TfoX and TfoY. *Cell Rep* **15**, 951-58, doi:10.1016/j.celrep.2016.03.092 (2016).

19 Bao, Y., Lies, D. P., Fu, H. & Roberts, G. P. An improved Tn7-based system for the single-copy insertion of cloned genes into chromosomes of gram-negative bacteria. *Gene* **109**, 167-8, doi:10.1016/0378-1119(91)90604-a (1991).

20 Meibom, K. L. *et al.* The *Vibrio cholerae* chitin utilization program. *Proc Natl Acad Sci U S A* **101**, 2524-9, doi:10.1073/pnas.0308707101 (2004).

21 Boyd, C. M. *et al.* Bacteriophage ICP1: A Persistent Predator of *Vibrio cholerae*. *Annu Rev Virol* **8**, 285-304, doi:10.1146/annurev-virology-091919-072020 (2021).

22 Seed, K. D. *et al.* Evidence of a dominant lineage of *Vibrio cholerae*-specific lytic bacteriophages shed by cholera patients over a 10-year period in Dhaka, Bangladesh. *mBio* **2**, e00334-10, doi:10.1128/mBio.00334-10 (2011).

23 Angermeyer, A., Das, M. M., Singh, D. V. & Seed, K. D. Analysis of 19 Highly Conserved *Vibrio cholerae* Bacteriophages Isolated from Environmental and Patient Sources Over a Twelve-Year Period. *Viruses* **10**, doi:10.3390/v10060299 (2018).

24 LeGault, K. N. *et al.* Temporal shifts in antibiotic resistance elements govern phage-pathogen conflicts. *Science* **373**, doi:10.1126/science.abg2166 (2021).

25 Alam, M. T. *et al.* Emergence and Evolutionary Response of *Vibrio cholerae* to Novel Bacteriophage, Democratic Republic of the Congo. *Emerg Infect Dis* **28**, 2482-90, doi:10.3201/eid2812.220572 (2022).

26 Seed, K. D. *et al.* Evolutionary consequences of intra-patient phage predation on microbial populations. *Elife* **3**, e03497, doi:10.7554/eLife.03497 (2014).

27 O'Hara, B. J., Alam, M. & Ng, W. L. The *Vibrio cholerae* Seventh Pandemic Islands act in tandem to defend against a circulating phage. *PLoS Genet* **18**, e1010250, doi:10.1371/journal.pgen.1010250 (2022).

28 Bhandare, S. G. *et al.* Complete Genome Sequences of *Vibrio cholerae*-Specific Bacteriophages 24 and X29. *Genome Announc* **5**, doi:10.1128/genomeA.01013-17 (2017).

29 Maffei, E. *et al.* Systematic exploration of *Escherichia coli* phage-host interactions with the BASEL phage collection. *PLoS Biol* **19**, e3001424, doi:10.1371/journal.pbio.3001424 (2021).
