# Supplementary material for: West African–South American pandemic Vibrio cholerae encodes multiple distinct phage defence systems
Source: Nat Microbiol. 2025 May 22;10(6):1352–65. doi: 10.1038/s41564-025-02004-9 (PMC12137116; doi:10.1038/s41564-025-02004-9)
Supplement: Supplementary file 2 — Reporting Summary [file 41564_2025_2004_MOESM2_ESM.pdf]

nature portfolio

Corresponding author(s): Melanie Blokesch
David W. Adams

Last updated by author(s): Mar 19, 2025

Reporting Summary

Nature Portfolio wishes to improve the reproducibility of the work that we publish. This form provides structure for consistency and transparency in reporting. For further information on Nature Portfolio policies, see our Editorial Policies and the Editorial Policy Checklist.

Statistics

For all statistical analyses, confirm that the following items are present in the figure legend, table legend, main text, or Methods section.

n/a	Confirmed	
☐	☒	The exact sample size (*n*) for each experimental group/condition, given as a discrete number and unit of measurement
☐	☒	A statement on whether measurements were taken from distinct samples or whether the same sample was measured repeatedly
☒	☐	The statistical test(s) used AND whether they are one- or two-sided<br>*Only common tests should be described solely by name; describe more complex techniques in the Methods section.*
☒	☐	A description of all covariates tested
☐	☒	A description of any assumptions or corrections, such as tests of normality and adjustment for multiple comparisons
☐	☒	A full description of the statistical parameters including central tendency (e.g. means) or other basic estimates (e.g. regression coefficient) AND variation (e.g. standard deviation) or associated estimates of uncertainty (e.g. confidence intervals)
☒	☐	For null hypothesis testing, the test statistic (e.g. *F*, *t*, *r*) with confidence intervals, effect sizes, degrees of freedom and *P* value noted<br>*Give P values as exact values whenever suitable.*
☒	☐	For Bayesian analysis, information on the choice of priors and Markov chain Monte Carlo settings
☒	☐	For hierarchical and complex designs, identification of the appropriate level for tests and full reporting of outcomes
☒	☐	Estimates of effect sizes (e.g. Cohen's *d*, Pearson's *r*), indicating how they were calculated

Our web collection on statistics for biologists contains articles on many of the points above.

Software and code

Policy information about availability of computer code

Data collection	Commercial software used for data collection was Zeiss Zen software (v.2.6 blue edition)
Data analysis	Commercial software used for data analysis was SnapGene v.4.3.11, Geneious Prime v.11.0.14.1, and LightCycler 96 software v.1.1.0.1320 (Roche). Open access software used for data analysis was ImageJ (version 2.1.0/1.53h; imagej.net/software/fiji), DefenseFinder v.1.2.4 Webserver (https://defensefinder.mdmlab.fr), PADLOC v.2.0.0 Webserver (https://padloc.otago.ac.nz/padloc/), MOTIF Search tool (genome.jp/tools/motif/), Jalview v.2.11.4.0, HHpred Webserver (https://toolkit.tuebingen.mpg.de/tools/hhpred; PDB_mmCIF70_8_Mar, default database), DALI Webserver (http://ekhidna2.biocenter.helsinki.fi/dali/), AlphaFold3 Webserver (https://alphafoldserver.com), Weblogo 3 Webserver (https://weblogo.threeplusone.com), clinker v.0.0.28, MacSyFinder v.2.1.1, MAFFT v.7.508, HMMER suite v.3.3.2, Unicycler v.0.5.0, snp-sites v.2.5.1, NCBI tool suite v.2.14.1, and flye v 2.9.3.

For manuscripts utilizing custom algorithms or software that are central to the research but not yet described in published literature, software must be made available to editors and reviewers. We strongly encourage code deposition in a community repository (e.g. GitHub). See the Nature Portfolio guidelines for submitting code & software for further information.

Data

Policy information about availability of data

All manuscripts must include a data availability statement. This statement should provide the following information, where applicable:

- Accession codes, unique identifiers, or web links for publicly available datasets
- A description of any restrictions on data availability
- For clinical datasets or third party data, please ensure that the statement adheres to our policy

The genome assemblies of V. cholerae strain N19-2759 have been deposited in the NCBI GenBank database under accession number GCA_046097525.1. The raw reads are available from the Sequence Read Archive (SRA) under submission number SRX26909066. The publicly available datasets used in this work and the dates that they were accessed are listed in the methods. All other data are available in the main text or the supplementary materials. Source data are provided with this paper.

Research involving human participants, their data, or biological material

Policy information about studies with human participants or human data. See also policy information about sex, gender (identity/presentation), and sexual orientation and race, ethnicity and racism.

Reporting on sex and gender	n/a (study on bacteria)
Reporting on race, ethnicity, or other socially relevant groupings	n/a (study on bacteria)
Population characteristics	n/a (study on bacteria)
Recruitment	n/a (study on bacteria)
Ethics oversight	n/a (study on bacteria)

Note that full information on the approval of the study protocol must also be provided in the manuscript.

Field-specific reporting

Please select the one below that is the best fit for your research. If you are not sure, read the appropriate sections before making your selection.

☒ Life sciences ☐ Behavioural & social sciences ☐ Ecological, evolutionary & environmental sciences

For a reference copy of the document with all sections, see nature.com/documents/nr-reporting-summary-flat.pdf

Life sciences study design

All studies must disclose on these points even when the disclosure is negative.

Sample size	All experiments were performed independently three times, unless explicitly stated otherwise. No statistical methods were used to predetermine sample size. The use of multiple independent biological repeats to ensure reproducibility is standard practice in the field.
Data exclusions	No data were excluded from the analysis.
Replication	All experimental data are representative of the results of three independent biological repeats, except for the BASEL bacteriophage collection screening, which was repeated twice. All replication attempts were successful.
Randomization	Randomization is not standard practice in the field and was not employed.
Blinding	Blinding is not standard practice in the field and was not employed.

Reporting for specific materials, systems and methods

We require information from authors about some types of materials, experimental systems and methods used in many studies. Here, indicate whether each material, system or method listed is relevant to your study. If you are not sure if a list item applies to your research, read the appropriate section before selecting a response.

Materials & experimental systems

n/a	Involved in the study
☐	☒ Antibodies
☒	☐ Eukaryotic cell lines
☒	☐ Palaeontology and archaeology
☒	☐ Animals and other organisms
☒	☐ Clinical data
☒	☐ Dual use research of concern
☒	☐ Plants

Methods

n/a	Involved in the study
☒	☐ ChIP-seq
☒	☐ Flow cytometry
☒	☐ MRI-based neuroimaging

Antibodies

Antibodies used

Primary antibodies against WonA (2210455), WonB (2210453), and VcSduA (2310059) were custom-raised in rabbits against synthetic peptides (Eurogentec) and used at a dilution of 1:500.

Direct-Blot™ HRP anti-E. coli RNA Sigma 70 Antibody (663205, BioLegend, U.S.A) was used at a dilution of 1:20,000.

The Anti-Rabbit IgG (whole molecule)–Peroxidase secondary antibody (A9169, Sigma-Aldrich) was used at a dilution of 1:20,000.

Validation

The specificity of custom-raised antibodies was validated by the inclusion of negative control samples lacking the relevant proteins in the Western Blot analysis.

For the commercial secondary antibody the following validation statement was taken from the Sigma website: Specificity of the Peroxidase Conjugated Anti-Rabbit IgG antibodies was determined by immunoelectrophoresis (IEP) versus normal rabbit serum and rabbit IgG.

For the commercial Direct-Blot™ HRP anti-E. coli RNA Sigma 70 Antibody the following validation statement was taken from the BioLegend website: Each lot of this antibody is quality control tested by Western blotting.

Plants

Seed stocks

Report on the source of all seed stocks or other plant material used. If applicable, state the seed stock centre and catalogue number. If plant specimens were collected from the field, describe the collection location, date and sampling procedures.

Novel plant genotypes

Describe the methods by which all novel plant genotypes were produced. This includes those generated by transgenic approaches, gene editing, chemical/radiation-based mutagenesis and hybridization. For transgenic lines, describe the transformation method, the number of independent lines analyzed and the generation upon which experiments were performed. For gene-edited lines, describe the editor used, the endogenous sequence targeted for editing, the targeting guide RNA sequence (if applicable) and how the editor was applied.

Authentication

Describe any authentication procedures for each seed stock used or novel genotype generated. Describe any experiments used to assess the effect of a mutation and, where applicable, how potential secondary effects (e.g. second site T-DNA insertions, mosiacism, off-target gene editing) were examined.
