# Supplementary material for: West African–South American pandemic Vibrio cholerae encodes multiple distinct phage defence systems
Source: Nat Microbiol. 2025 May 22;10(6):1352–65. doi: 10.1038/s41564-025-02004-9 (PMC12137116; doi:10.1038/s41564-025-02004-9)

Source Data Fig. 3E - Western blot of WonB levels over time

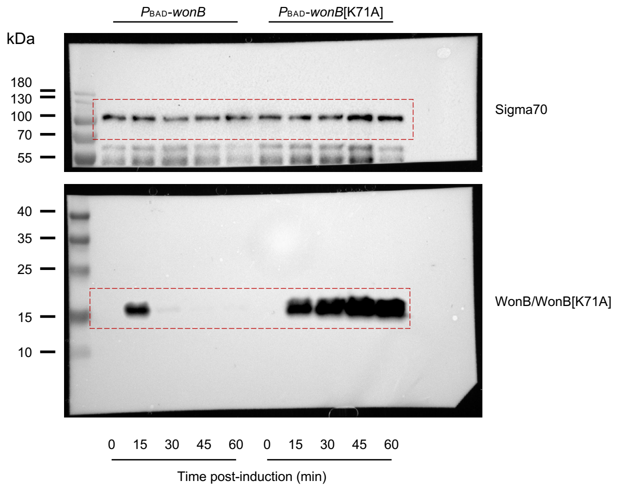

Source Data Fig. 3F - Western blot of WonB stability over time

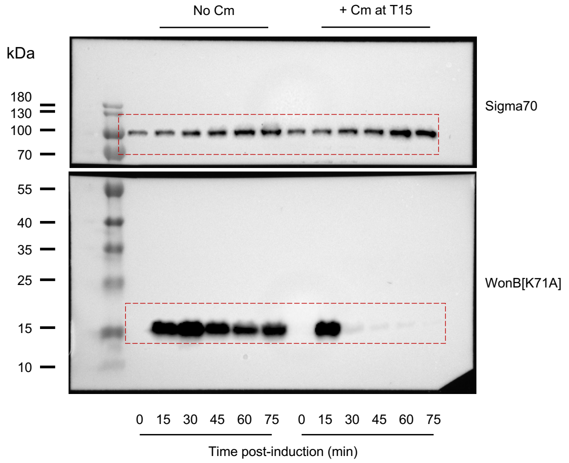

Source Data Fig. 3G - Western blot comparing WonB stability in *lon*+ vs Δ*lon*

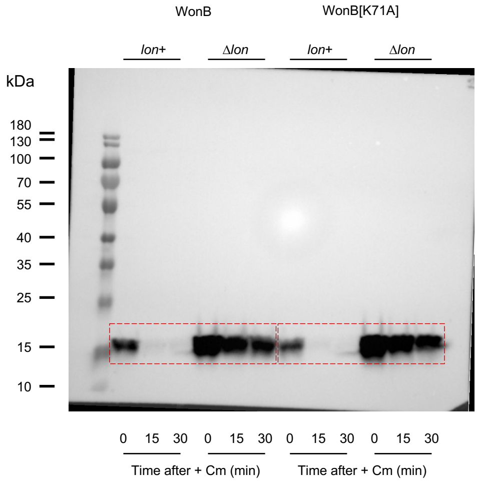

Source Data Extended Data Fig. 3E - Western blots of WonA and WonB

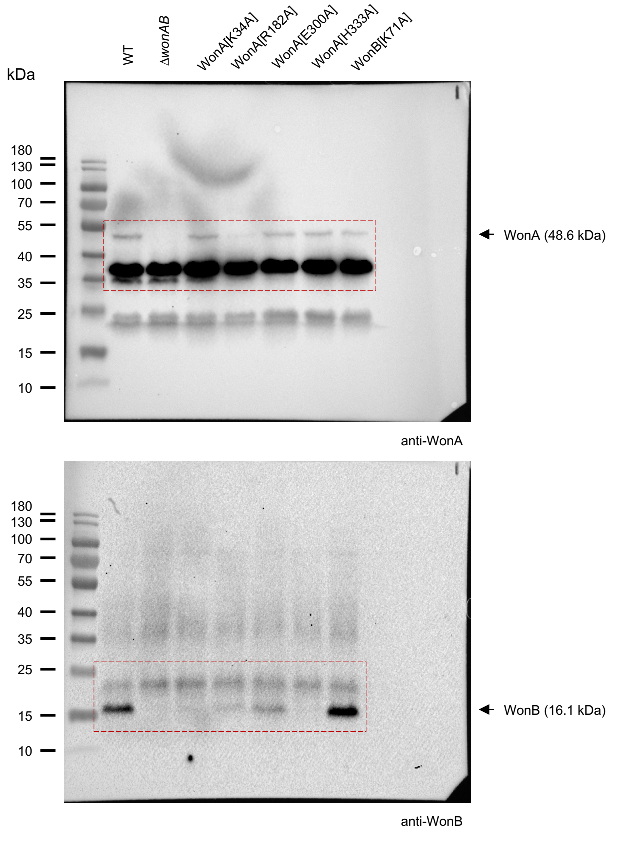

Source Data Extended Data Fig. 6C - Western blots of WonA and WonB levels

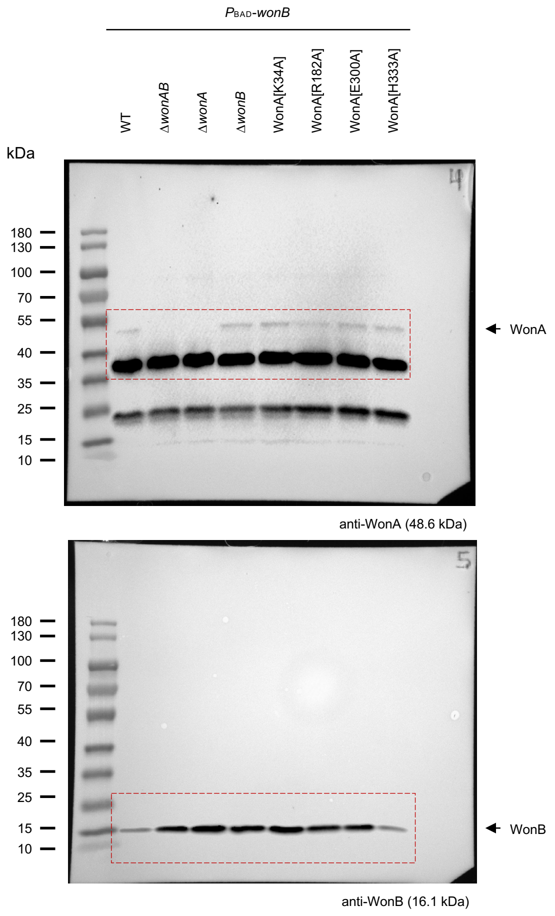

Source Data Extended Data Fig. 6E - Agarose gels of genomic DNA extractions

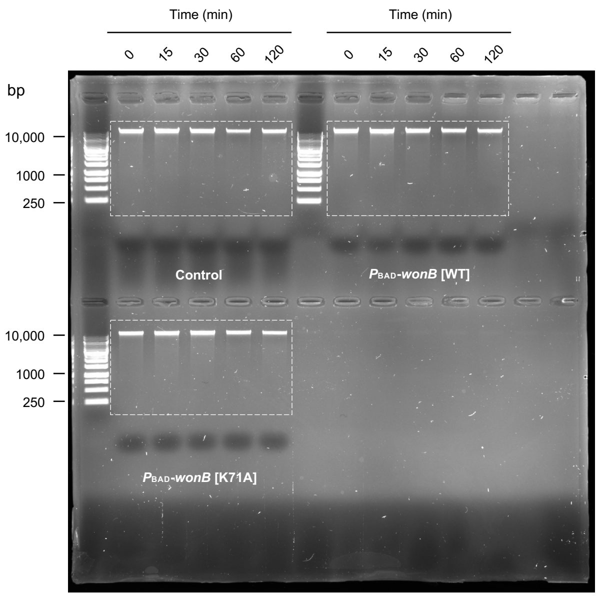

Source Data Extended Data Fig. 10D - Western blot of *Vc*SduA

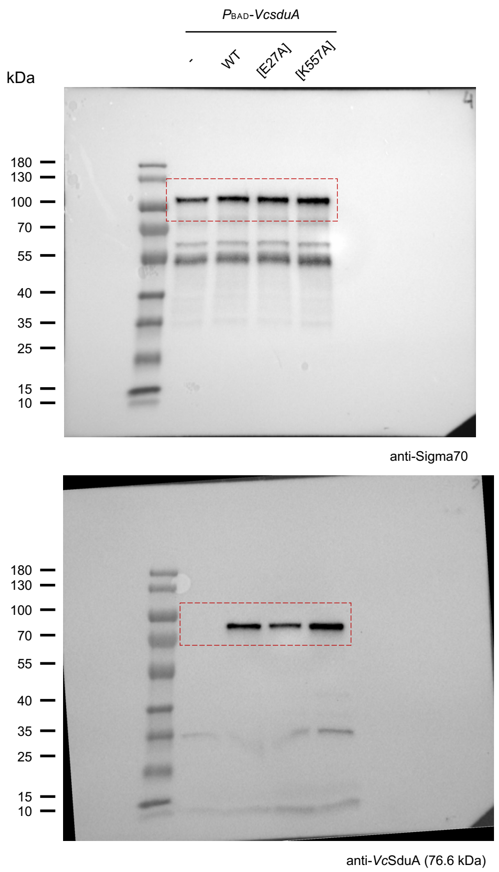

Supplement: Supplementary file 17 — Unprocessed western blots and gels. [file 41564_2025_2004_MOESM17_ESM.pdf]
